# A New Energy Approach to Predicting Fracture Resistance in Metals

**DOI:** 10.3390/ma16041566

**Published:** 2023-02-13

**Authors:** Maciej Dutkiewicz, Oksana Hembara, Olha Chepil, Mykhailo Hrynenko, Taras Hembara

**Affiliations:** 1 Faculty of Civil and Environmental Engineering and Architecture, Bydgoszcz University of Science and Technology, 85-796 Bydgoszcz, Poland; 2 Karpenko Physico-Mechanical Institute, National Academy of Sciences of Ukraine, 79601 Lviv, Ukraine; 3 Institute of Civil Engineering and Building Systems, Lviv Polytechnic National University, 79000 Lviv, Ukraine; 4 The Edicational Scentific Institute of Civil Protection, Lviv State University of Life Safety, 79000 Lviv, Ukraine

**Keywords:** true stress–strain curves, fracture energy, hydrogen concentration, pre-strain, hydrogen embrittlement

## Abstract

**Highlights:**

**What are the main findings?**

- A new approach to predicting fracture resistance is proposed.
- A correlation was established between the surface and volume stress-deformed states.

**What is the implication of the main finding?**

- The stress-strain state of the modified Bridgman sample under four load modes was investigated using two methods: nu-merical and experimental.
- The fracture energy and the damage parameter were established as characteristics of resistance to fracture.

**Abstract:**

To ensure the reliability and durability of structural elements, modern approaches require data characterizing the local stress-strain state of the material in risk zones. In order to predict the fracture resistance of structural elements, a theoretical-experimental method based on the damage accumulation model using the energy approach is proposed. One of the unique characteristics of the proposed approach is that it uses local parameters of the stress-strain state, which are determined using a highly accurate and easy-to-use noncontact method of optical–digital image correlation (ODIC). This can be used both in laboratory conditions and for structural elements under real operating conditions. The proposed method of plotting stress–strain curves makes it possible to determine the true stresses near the concentrators in structural elements under a complex load. Using these diagrams in calculations and the finite element method (FEM), a study of local strain not only of the surface, but also of the internal volumes of the material was carried out. The damage parameter is introduced as the ratio of the elastoplastic strain energy of the local volume to its critical value. It is confirmed that the damage of the sample material starts from its centre. It was established that the damage parameter in the centre of the sample is 25–35% higher than its value on the surface of the sample.

## 1. Introduction

In most cases, the operation of structural elements under working conditions leads to their exposure to the complex action of the force load and the working environment for an extended period. In particular, nuclear and thermal energy structure elements work under thermal force loading and the impact of a hydrogen-containing environment [1,2]. As a result, the stress-strain state of the material changes under the influence of these factors, which affects the resource of its work [3]. The study of the mutual impact of these factors on the change of the stress-strain state in the metal and the work resource of structural elements is relevant, both from a scientific and a practical point of view. As a result of the operation, several mechanisms of damage to structural materials appear simultaneously [4,5]. The resource of equipment elements of thermal and nuclear power plants is traditionally defined as the smallest resource when taking into account each of these damage mechanisms. At the same time, naturally, the mutual influence of various damage mechanisms on the resource is neglected. Such a traditional approach can lead to both underestimated and overestimated lifetime of equipment elements, which leads to economic costs in the operation of thermal and nuclear power plants, for example, in the case of unforeseen violations of normal operation or replacement of equipment elements that can actually be operated for quite a long time.

Objective information about material properties can be obtained based on the stress-strain dependencies, i.e., stress–strain curves [6,7]. The accuracy of the established dependencies determines the reliability of engineering calculations of the strength and the reliability of structural elements. It is quite challenging to establish the stress-strain state in the vicinity of stress concentrators under significant plastic strain. Within the framework of fracture mechanics, theoretical and experimental approaches have been developed, which make it possible to directly or indirectly solve this problem [8]. In particular, a method was introduced and tested to plot stress–strain curves to establish the dependencies between stresses and strains based on the high-precision determination of local strain by optical–digital methods.

Among the modern optical–digital methods of measuring displacements, the optical–digital image correlation method is one of the most promising [9].

The ODIC method has gained recognition [10,11]. It is widely used as a powerful and flexible tool for determining surface strain in the experimental mechanics of a deformed solid body. It provides a wide field for the determination of displacements and strain in the measurement area thanks to its comparison of digital images of the sample surface in undeformed and deformed states [12,13]. This optical method is based on digital processing according to the appropriate algorithm of several images of the sample surface, which are obtained with known values of the load force using a camera [14]. As each subsequent image is compared to the previous one, the movement of the surface within the fragment is determined. After relating the amount of movement to the size of the fragment, the strain value is set [15,16].

**Figure 1:**
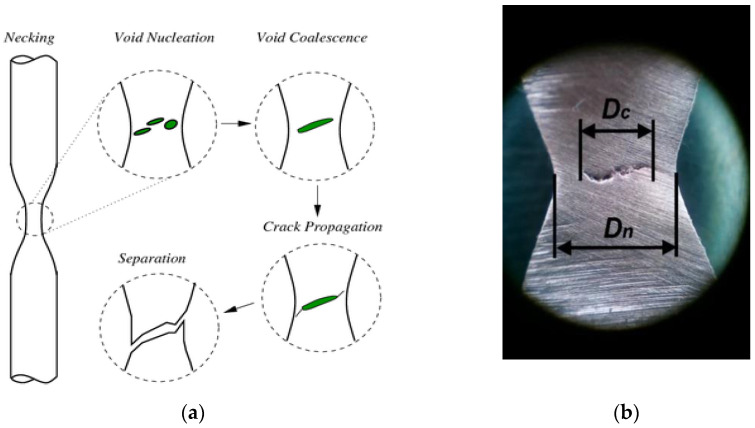
Scheme of damage formation (**a**) and internal crack in the sample neck (**b**) [17].

However, this method makes it possible to obtain the field of displacements and strain only over the sample surface, and it is known that fractures usually begin at the centre of the sample. (Figure 1) [17].

With the introduction of the ODIC method into the experimental and FEM into the theoretical mechanics of materials, it became possible to precisely study the local strain, not only on the surface but also on the internal volumes of the material. Experimental studies [8,18] showed that the damage accumulation and fracture of cylindrical steel samples under static tension begin in the internal volumes of the material and not from the surface. This is due to the inhomogeneity of the stress-strain state in the zone of neck formation [19,20]. This inhomogeneity depends on the applied load, the prestrain, and the influence of the environment [21,22,23]. This can be investigated using a combination of experimental testing and theoretical modelling. Damage accumulation models are widely used in fatigue analysis of engineering structures. A review article [24] provided a comprehensive overview of the state-of-the-art in cumulative damage and lifetime prediction models for the endurance-based high-cycle fatigue design of metal structures. Existing damage accumulation models for general multiaxial loadings can be classified as stress-based [25,26,27,28,29], strain-based [30,31,32], energy-based [33,34,35,36,37], fracture mechanics-based [38,39], and other methods [40,41]. The damage propagation models for energy-based are also used for composites [42,43]. The authors [44,45] developed an energy-based damage model for the prediction of the damage and postdamage initiations as well as the subsequent softening process of composite laminate materials [45,46,47,48]. 

The purpose of this work was to develop a modern nonfracture approach for assessing the fracture resistance of structural elements based on the determination of the local stress-strain state of the material in risk zones. In this study, an energy approach is developed based on the elastoplastic strain energy dissipation of a modified Bridgman specimen using a damage model to estimate the fracture resistance in metals under complex loading. The critical value of the energy of elastoplastic strain (fracture energy) is accepted as a characteristic of the material’s fracture resistance under given conditions of load, temperature, and working environment. Continuum damage mechanics, which consider the initiation and propagation of damage at a material point, are used to quantify the material softening process and calculate the damage evolution parameter. The damage parameter is entered as the ratio of the elastoplastic strain energy of the local volume to its critical value. The evolution of the energy of elastoplastic strain is established using FE modelling of the Bridgman sample under conditions simulating experimental tests in order to establish the relationship between the surface and the bulk stress-strain state of the sample. The fracture energy is determined experimentally as the area under the “stress–strain” curve, where the true strain to the local volume is calculated based on displacement data from two directions on the basis of 0.5 mm with the use of the ODIC method. The value of the true stresses in the zone of maximum strain is determined by taking into account the change in the cross-sectional area caused by the change in the diameter of the sample during its deformation by uniaxial tension. The fracture energy and the damage parameter for the modified Bridgman sample made of 22K steel under different load regimes were determined theoretically and experimentally.

This method is recommended for determining the local stress-strain state of metal structures and predicting the accumulation of damage in them under various types of loading. Strain measurement during the testing is realized using the ODIC technique. This allows us to determine local strains and calculate the strain energy density absorbed by the material until the moment of fracture. The nonfracture ODIC technique can be also used for verification of the calculated results and the stress-strain state determination of metal structures during the full-scale testing.

## 2. Numerical Simulations

### 2.1. Energy Approach for Modelling Damage Accumulation

The approach based on the elastoplastic strain energy is promising. The energy approach directly takes into account the entire history of stress and strain changes to calculate the durability of a structural element. The energy approach also takes into account the fundamental response of the material and can directly correlate energy with damage from external loading. In the process of plastic strain, until the moment of fracture, the material absorbs the energy supplied to it from the outside. When the energy reaches a critical value in the local volume of the material, a microcrack of critical size appears, which results in a sharp localization of strain and fracture. Thus, the fracture criterion is the condition for reaching the critical value of the specific strain energy, i.e., the specific fracture energy [49], as follows:

(1) $$W(P_{*},T_{*},C_{H*})=W_{C},$$

where *W* is the strain energy of the local volume of the element, calculated using the FEM; $P_{*},T_{*},C_{H*}$ is the pressure, temperature, and hydrogen concentration, at which the strain energy becomes critical; and $W_{C}$ is the characteristic fracture resistance of a material under given conditions of loading, temperature, and working environment, which is determined experimentally using the ODIC method.

The total specific energy required for the fracture of a metal unit volume consists of the elastic strain energy, the plastic strain energy, and the crack movement energy. In this case, the plastic strain energy is much higher than other components. It was shown in [34] that the energy absorbed by a unit’s volume during deformation to the fracture can be determined by the area under the curve, which is « true stress *S_i_*, -true strain *e_i_*. 

The ratio of the elastoplastic strain energy of the local volume to its critical value is used as the measure of damage [40]:

(2) $$\omega =W/W_{C}.$$

The fracture of the volume element of the material will occur due to achieving equality as follows:

(3) ω=1.

### 2.2. Rate-Independent Plasticity

Material nonlinearities occur because of the nonlinear relationship between stress and strain; that is, the stress is a nonlinear function of the strain.

***Strain Definitions***. In the case of nonlinear materials, the definition of total strain has the form:

(4) $$\left\{\varepsilon^{tot}\right\}=\left\{\varepsilon^{el}\right\}+\left\{\varepsilon^{pl}\right\},$$

where $\left\{\varepsilon^{tot}\right\}$ is the total strain vector, $\left\{\varepsilon^{el}\right\}$ is the elastic strain vector, and $\left\{\varepsilon^{pl}\right\}$ is the plastic strain vector.

***Yield Criterion.*** The yield criterion determines the stress level at which yielding is initiated. For multicomponent stresses, this is represented as a function of the individual components, $F\left(\left\{\sigma \right\}\right)$, which can be interpreted as an equivalent stress $\sigma_{e}$:

(5) $$\sigma_{e}=F\left(\left\{\sigma \right\}\right),$$

where $\left\{\sigma \right\}$ is the stress vector.

When the equivalent stress is equal to a material yield parameter $\sigma_{pl}$,

(6) $$F\left(\left\{\sigma \right\}\right)=\sigma_{pl},$$

the material will develop plastic strains. If $\sigma_{e}$ is less than $\sigma_{pl}$, the material is elastic, and the stresses will develop according to the elastic stress–strain relations.

The flow rule determines the direction of plastic straining and is given as:

(7) $$\left\{d\varepsilon^{pl}\right\}=\lambda \left\{\partial Q^{pl}/\partial \sigma \right\},$$

where λ is the plastic multiplier (which determines the amount of plastic straining) and $Q^{pl}$ is where the function of stress is termed the plastic potential (which determines the direction of plastic straining).

If *Q^pl^* is the yield function (as is normally assumed), the flow rule is termed associative, and the plastic strains occur in a direction normal to the yield surface.

Using the hardening rule used in [50] obtained:

$$\lambda =\frac{\left\{\frac{\partial F}{\partial \sigma }\right\}\left[M\right]\left[D\right]\left\{d\varepsilon^{tot}\right\}}{-\left\{\frac{\partial F}{\partial \kappa }\right\}{\left\{\sigma \right\}}^{T}\left[M\right]\left\{\frac{\partial Q^{pl}}{\partial \sigma }\right\}-C{\left\{\frac{\partial F}{\partial \sigma }\right\}}^{T}\left[M\right]\left\{\frac{\partial Q^{pl}}{\partial \sigma }\right\}+{\left\{\frac{\partial F}{\partial \sigma }\right\}}^{T}\left[M\right]\left[D\right]\left\{\frac{\partial Q^{pl}}{\partial \sigma }\right\}},$$

where [*M*] is the diagonal matrix with $a_{11}=a_{22}=a_{33}=1,\;a_{44}=a_{55}=a_{66}=2\;$; *C* is the material parameter; and [*D*] is the stress-strain matrix.

The size of the plastic strain increment is therefore related to the total increment in strain, the current stress state, and the specific forms of the yield and potential surfaces. The plastic strain increment is then calculated using (7).

### 2.3. The Strain Energy Determination

The principle of virtual work states that a virtual (very small) change to the internal strain energy must be offset by an identical change in external work due to the applied loads, or:

(8) δW=δA,

where W is the strain energy (internal work), A is the external work, and δ is the virtual operator.

The virtual strain energy is:

(9) $$\delta W=\int_{V_{e}}\left\{\delta \varepsilon^{tot}\right\}\left\{\sigma \right\}d{\left(V_{e}\right)}^{T},$$

where $V_{e}$ is the volume of the element.

To determine the distribution of strain energy, Equation (9) was solved, where stresses and strains are determined based on the solution of the elastic–plastic–temperature problem using the finite element method.

The coupled-field finite element matrix equation for the thermoplastic analysis is:

$$\left[\begin{array}{cc} \left[M\right] & \left[0\right]\\ \left[0\right] & \left[0\right] \end{array}\right]\left\{\begin{array}{c} \left\{\ddot{u}\right\}\\ \left\{\ddot{T}\right\} \end{array}\right\}+\left[\begin{array}{cc} \left[C\right] & \left[0\right]\\ \left[0\right] & \left[C^{t}\right] \end{array}\right]\left\{\begin{array}{c} \left\{\dot{u}\right\}\\ \left\{\dot{T}\right\} \end{array}\right\}+\left[\begin{array}{cc} \left[K\right] & \left[0\right]\\ \left[0\right] & \left[K^{t}\right] \end{array}\right]\left\{\begin{array}{c} \left\{u\right\}\\ \left\{T\right\} \end{array}\right\}=\left\{\begin{array}{c} \left\{F\right\}\\ \left\{Q\right\}+\left\{Q^{pl}\right\} \end{array}\right\}$$

where

[*M*] = element mass matrix;

[*C*] = element structural damping matrix;

[*K*] = element stiffness matrix;

{*u*} = displacement vector;

{*F*} = sum of the element nodal force and element pressure vectors;

[*C^t^*] = element-specific heat matrix;

[*K^t^*] = element diffusion conductivity matrix;

{*T*} = temperature vector;

{*Q*} = sum of the element heat generation rate load and element convection surface heat flow vectors; and

$\left\{Q^{pl}\right\}$ = element plastic heat generation rate load (7).

### 2.4. Model of the Modified Bridgman Sample

All numerical simulations presented in this work were conducted using the explicit finite element solver ANSYS 2022R1 [50]. In particular, the Static Structural solver from the Workbench 2022R1 platform was used.

**Figure 2:**
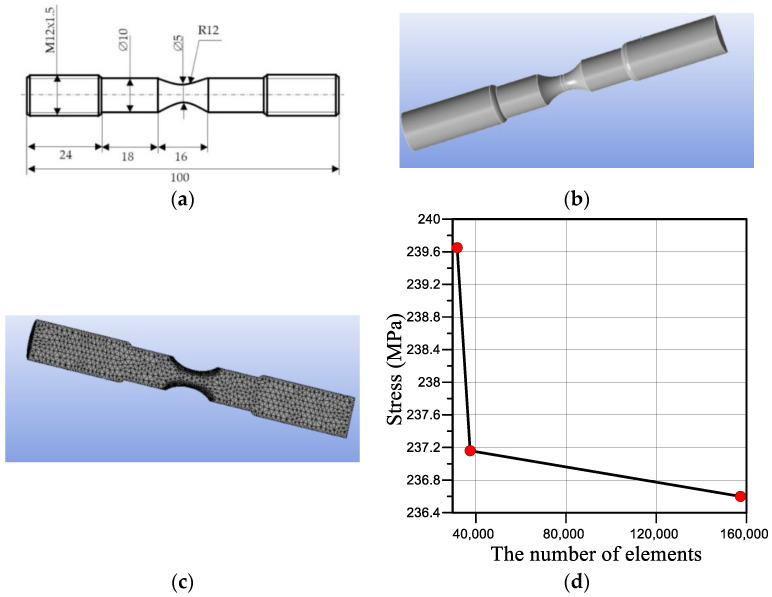
Schematic illustration of the geometry (**a**), the sample view (**b**), the sample breakdown into finite elements (**c**), and the mesh convergence study (**d**).

Calculations were performed for the modified Bridgman sample made of steel 22K (Figure 2a), which was tested experimentally in other work [18]. Taking into account the symmetry of the sample, a three-dimensional calculation model (Figure 2b) was built for half of the sample relative to the longitudinal plane of symmetry (Figure 2c). To ensure sufficient accuracy in calculations of the area of large strain, while constructing the model, the size of the elements was reduced in the direction from the gripping to the working part of the sample. The finite element mesh, which was denser in the vicinity of the neck of the sample, contains 132,345 nodes and 31,752 tetrahedron-like elements. During the calculations, the convergence of the partition grid was carried out, and it was established that when the number of elements increases by four times, the obtained result will change by only 1.23% (Figure 2d). The obtained results confirm the sufficiency of the number of elements required to obtain reliable results.

**Figure 3:**
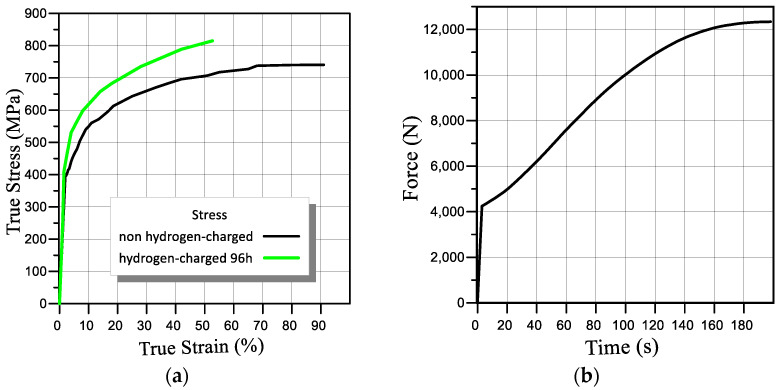
Stress–strain curves of steel 22K (**a**) and applied load (**b**).

In order to take into account the material’s plasticity, during the Engineering Date module, the material plasticity characteristic Multilinear Isotropic Hardening was added to the steel properties, into which the experimentally obtained strain-stress dependence was entered (Figure 3a). To implement axial tensile loading of the model, the sample was fixed on one side, and a time-varying force equivalent to the load applied to the sample during the experiment was applied to the other side (Figure 3b).

**Figure 4:**
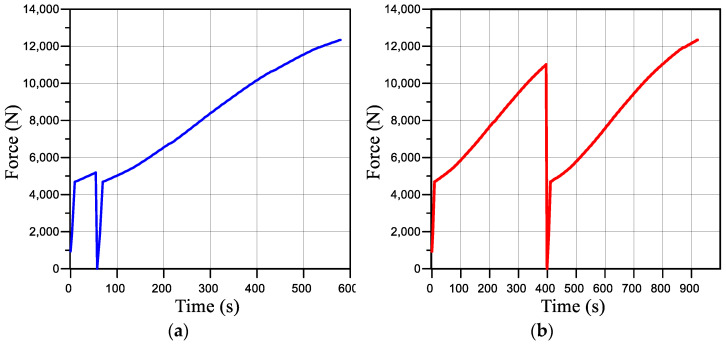
Sample loading diagram for modes II (**a**) and III (**b**).

Calculations were carried out for the four loading modes described in Section 3 (Figure 3 and Figure 4).

## 3. Experimental Procedures

A technique to plot stress-strain curves based on the non-contact ODIC method was used to determine true strains in the stress-strain homogeneous region of a sample. Additional information regarding the geometry, load modes, and materials of the modified Bridgman sample is described in detail in our previous paper [18]. Modified Bridgman cylindrical steel 22K samples were created to plot fracture diagrams (Figure 4a) using a quantity of three samples for different research parameters. The chemical composition (mass, %) of the material was: 0.17–0.24% C; 0.17–0.37% Si; 0.35–0.65% Mn; ≤0.035% P; ≤0.040% S; ≤0.25% Cr; ≤0.30% Cu; ≤0.30% Ni. Such a modified design of the Bridgman sample makes it possible to unambiguously determine the value and place of the local elastoplastic strain, the formation of a “neck,” and the fracture. For the correct application of the ODIC method, a base was applied with black paint to the specially prepared working surface of the samples. Next, a stochastic dot pattern was sprayed on this base using white paint.

According to this method, the value of the true strain *e_i_* to the local volume is calculated based on the displacement data from two directions on the basis of 0.5 mm using an optical–digital speckle image correlator [15]. The value of true stress *S_i_* for the area of maximum strain is determined using *S_i_ = P_i_/F_i_,* where *F_i_* is the current value of the cross-sectional area, which takes into account the change in its diameter.

The fracture energy $W_{C}$ was calculated by replacing the integration with the sum of elementary areas using the formula:

(10) $$W_{C}=\frac{1}{2}\sum_{i=0}^{i_{*}-1}\left(S_{i+1}+S_{i}\right)\left(e_{i+1}-e_{1}\right),$$

where i_*_ is the element number (or time step) at which the strain energy reaches its maximum value; $S_{i}$ is the value of the true stresses in the *i*-th element; and $e_{i}$ is the value of the true strain in the *i*-th element.

The fracture energy was determined experimentally (10) as the area under the “true stress–true strain” curve, where the true strains in the local volume were calculated based on displacement data from two directions on the basis of 0.5 mm using the ODIC (optical–digital image correlation) method. The value of the true stresses in the zone of maximum strain is determined by taking into account the change (necking) in the cross-sectional area resulting from the change in the diameter of the sample during its deformation by uniaxial tension.

The tests were carried out for thermal energy steel of type 22K, for which the yield strength was $\sigma_{t}=250\;\mathrm{MPa}$ and the ultimate strength was $\sigma_{B}=490\;\mathrm{MPa}$. In our previous work [18], the stress–strain curves of the modified Bridgman samples of this steel were plotted at different levels of prestrain and hydrogenation time using the ODIC method. In this article, we considered four load modes:

1. The tensile load to Pmax = 12,340 N.
2. The sample was preloaded to a force of 5200 N that resulted in stresses equal to the yield point in the sample, then unloaded, and after 2 min, it was reloaded. The loading scheme is presented in Figure 4a.
3. The sample was preloaded to a force of 10,950 N that caused stresses equal to the strength limit to occur in the sample, then unloaded, and after 2 min, it was reloaded again. The loading scheme is presented in Figure 4b.
4. The sample was prehydrogenated in an autoclave for 96 h and then was under tensile load.

The value of the fracture energy (Table 1) was determined for four load modes both experimentally on the surface of the sample and numerically in the centre.

## 4. Experimental-Computational Approach

**Figure 5:**
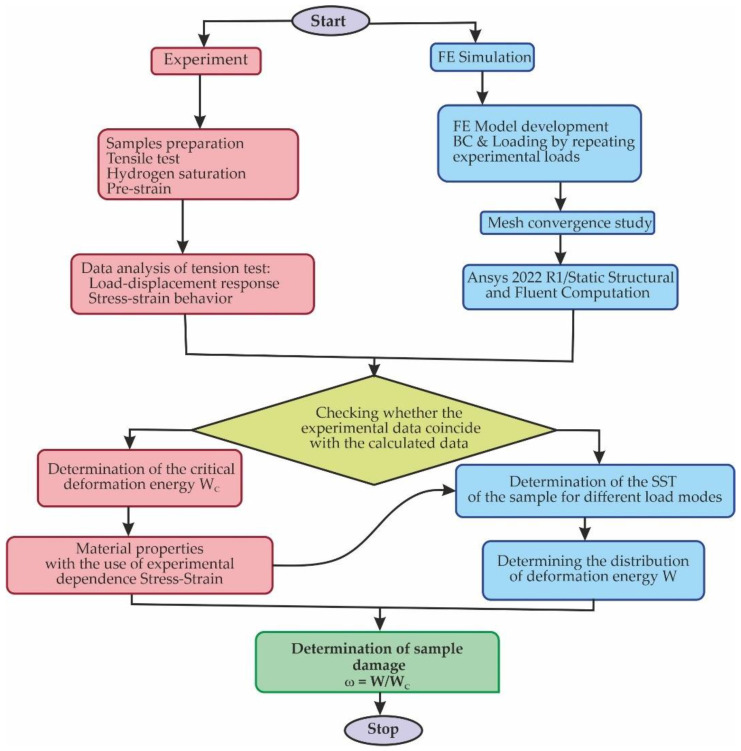
Block diagram of research methodology.

An experimental–computational approach was developed to study elastoplastic strain and damage evolution in steel under complex loading using a damage model based on an energy approach. A flowchart of the process is shown in Figure 5. This approach logically links experimental data with simulation results. In the first stage (experiment), a set of tests on the uniaxial tension of the modified Bridgman sample before failure was implemented. The stress–strain curves were plotted under different conditions of prestrain and hydrogenation. The fracture energy was determined for each loading mode.

In the second stage (FE simulation), a three-dimensional FE model of the Bridgman specimen was developed, which was used to estimate the energy of elastoplastic strain of steel and the evolution of damage accumulation not only on the surface but also on the internal volumes of the material.

## 5. Results and Discussion

**Figure 6:**
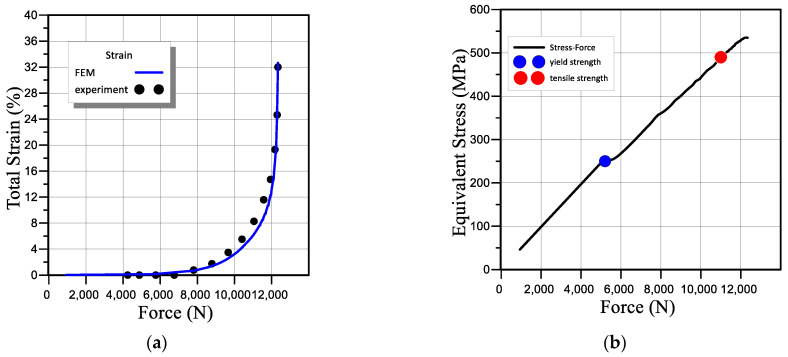
Comparison of calculated values of strain (**a**) and stress (**b**) using experimental data.

To verify the model, the experimentally established and obtained using FEM total strain depending on the applied force were compared (Figure 6a). We also plotted the dependence of the equivalent stresses on the applied force around the neck of the sample (where their values reach their maximum) (Figure 6b). This made it possible to establish the value of the applied load, at which the equivalent stresses reach the values $\sigma_{t}=250\;\mathrm{MPa}$ (yield stress for this steel in Figure 6b is marked with a blue dot) and $\sigma_{B}=490\;\mathrm{MPa}$ (ultimate stress for this steel in Figure 6b is indicated by a red dot). The comparison of experimental and calculated data shown in Figure 6 provides a satisfactory match.

**Figure 7:**
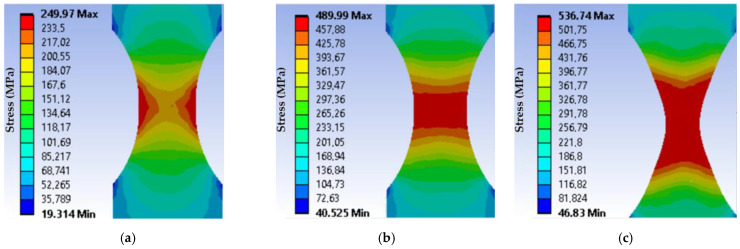
Equivalent stresses distribution in the sample during its loading under the load mode I: (**a**) when the equivalent stresses reach the yield strength; (**b**) when the equivalent stresses reach the ultimate strength; (**c**) at the fracture moment.

**Figure 8:**
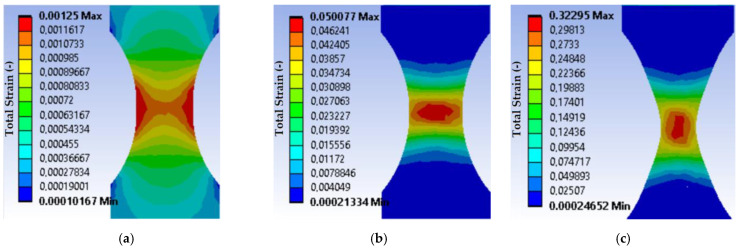
Total strain distribution in the sample during its loading under the load mode I: (**a**) when the equivalent stresses reach the yield strength; (**b**) when the equivalent stresses reach the ultimate strength; (**c**) at the fracture moment.

As a result, the distribution of the total strains, equivalent stresses, and strain energy along the thickness of the neck of the sample, starting from the centre, was obtained. Figure 7 and Figure 8 show the equivalent stress distributions and general elastoplastic strains in the sample during its loading under load mode I at different loading moments. The results of the calculations indicate a difference in the stress-strain state of the material in the centre and on the surface of the working part of the sample. Within the limits of elastic strain, its values decrease from the centre as they approach the sample edge. It should be noted that the strain gradient decreases with increasing force.

**Figure 9:**
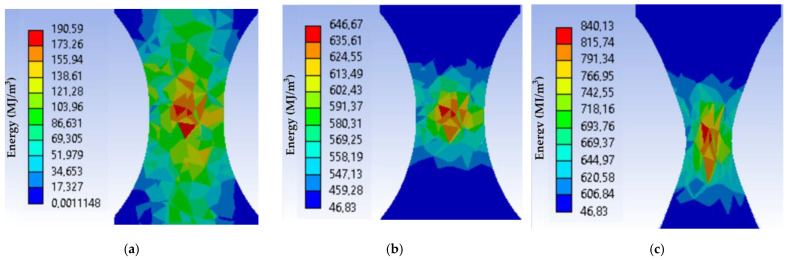
Strain energy distribution in the sample during its loading under the load mode I: (**a**) when the equivalent stresses reach the yield strength; (**b**) when the equivalent stresses reach the ultimate strength; (**c**) at the fracture moment.

Figure 9 shows the change in the elastoplastic strain energy at different moments of loading along the cross-section of the sample. The inhomogeneity of the stress-strain state along the cross section is very clearly visible here. We see that, at the moment of fracture, the maximum energy value is reached in the sample centre, which confirms the experimental results about the initiation of a crack inside the sample.

**Figure 10:**
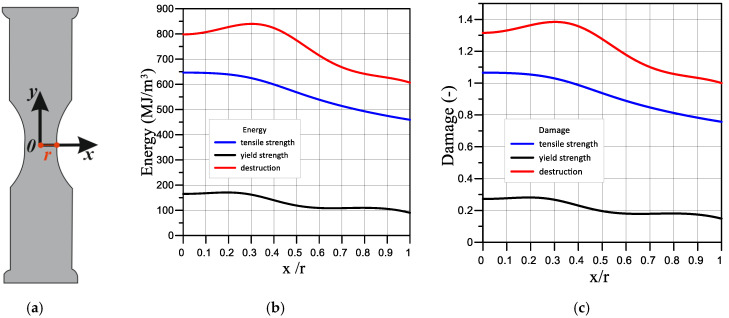
Diagram of the place where the results were obtained (**a**) Critical value distribution of the strain energy (**b**) and the damage parameter (**c**) in the sample under the load mode I: black curve—when the equivalent stresses reach the yield strength; blue—when the equivalent stresses reach the ultimate strength; red—at the fracture moment.

**Figure 11:**
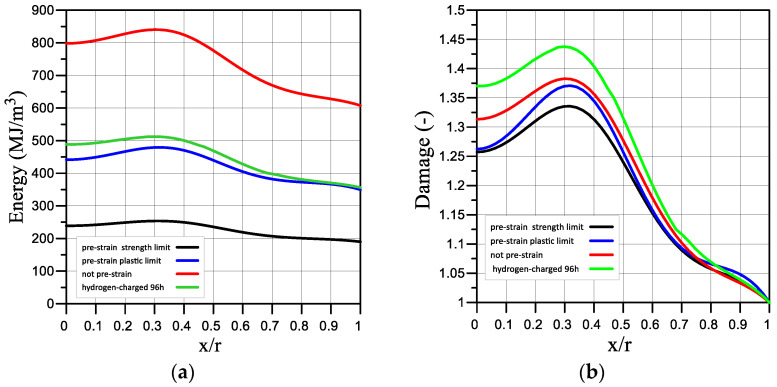
The critical value distribution of strain energy (**a**) and the damage parameter (**b**) in the sample under four load modes: not pre-strained—I mode (red), pre-strained to the yield strength—II mode (blue), pre-strained to the ultimate strength—III mode (black), pre-hydrogenated—VI mode (green).

Figure 10 and Figure 11 show the distribution of the critical value of the elastoplastic strain energy *W* (b) and the damage parameter (c) across the thickness of sample (a) under different loading modes. We see that the maximum values are reached in the sample centre, and the difference reaches up to 50%.

It was established that strain and fracture energy are characteristics that are sensitive to the load conditions. The experimentally determined value of the specific fracture energy of steel 22K in the initial state is equal to 607 MJ/m^3^ (load mode I). Prehydrogenation of the sample for 96 h reduces the fracture energy to 356 MJ/m^3^ (load mode IV), which is 41% of the loss of the fracture resistance of the sample. With prestrain up to 50% (load mode II) and 75% (load mode II) the fracture energy decreases to 349 MJ/m^3^ and 190 MJ/m^3^, which is 42% and 69% of the sample’s fracture resistance loss, respectively. It should be noted that these quantities, which represent the fracture energy, were defined as the area under the curve « true stress *S_i_*, -true strain *e_i_*. The value of the true strain *e_i_* in the local volume is calculated based on the displacement data from only the points of the sample surface in two directions. FEM calculations made it possible to obtain the critical value distribution of the strain energy and the damage parameter over the sample thickness (Figure 10 and Figure 11). Uneven distributions of these values were obtained for all load modes. At the same time, the fracture energy in the sample centre is significantly higher than its value obtained experimentally from the true stress-strain diagram. In particular, at the fracture moment (Figure 11a), the difference is 25% for load mode I, 20.8% for mode II, 20.5% for mode III, and 35.7% for mode IV.

In Figure 11a, we can see that the fracture resistance decreases the most in the sample that was prestrained to 75% (black curve). However, as can be seen in Figure 11b, the largest damage is achieved in the sample centre in the sample that was prehydrogenated (green curve). Moreover, the damage parameter is higher in the sample centre than on its surface for all load modes.

**Figure 12:**
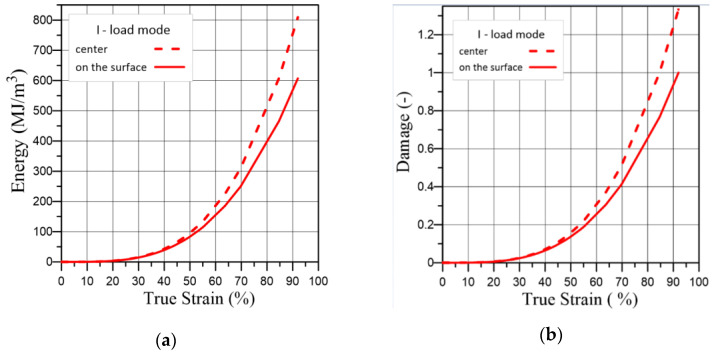

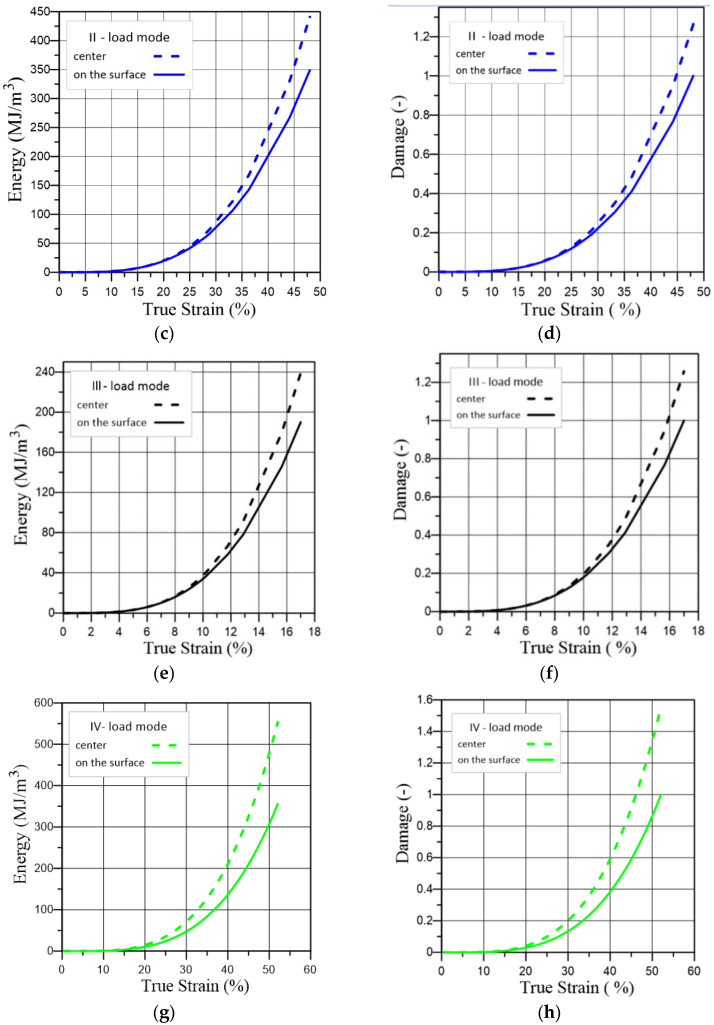
(**a**,**c**,**e**,**g**) Strain energy—local true strain response and (**b**,**d**,**f**,**h**) damage parameter—local true strain response.

We also plotted the dependence of the strain energy and the damage parameter in the sample centre (x = 0, Figure 10a) and on the surface (x = r, Figure 10a) using the local surface strain (Figure 12), which was measured using the ODIC method under four load modes: not prestrained (red), prestrained to the yield strength (blue), prestrained to the ultimate strength (black), and prehydrogenated (green).

We observed the same trend for all load modes; namely, the difference between the fracture energy and the damage parameter after a certain strain value. Therefore, when monitoring the stress-strain state of the surface using the ODIC method, it is necessary to take into account the inhomogeneity of the stress-strain state along with the thickness of the structural element and the possibility of the fracture resisting its various parts. As the results of this work show, at a safe level of the surface layers’ strain on a structural element, a fracture can already begin inside it.

## 6. Conclusions

A theoretical–experimental approach for predicting the accumulation of damage using an energy approach is proposed, which allows for the monitoring of the stress-strain state of a structural element in any local volume. The energy distribution of elastoplastic strain was determined using the finite element method. In the algorithm for solving the elastoplastic problem, a multiline diagram of the true strain under different load modes was used. The fracture energy was determined experimentally to be the area under the “true stress–true strain” curve, where the true strains in the local volume were calculated based on displacement data from two directions on the basis of 0.5 mm using the ODIC method. The value of the true stresses in the zone of maximum strain was determined by taking into account the change in the cross-sectional area caused by the change in the diameter of the sample during its deformation by uniaxial tension.

Experimental tests of the sample with uniaxial tension were reproduced with the help of FEM simulation. However, the ODIC method only allows results to be obtained from the surface of the sample. The use of three-dimensional FEM made it possible to obtain the distribution of stresses and strains throughout the volume of the sample, specifically, from the centre.

(1) FEM calculations of the stress-strain state and damage accumulation in the modified Bridgman sample under conditions reproducing experimental tests were carried out.
(2) It was established that there is a difference in the stress-strain state of the material in the centre and on the surface of the working part of the sample. Higher stress and strain values are reached in the sample centre.
(3) It was established that the fracture energy in the sample centre is significantly higher than its values obtained experimentally from the true stress-strain diagram. In particular, at the fracture moment, the difference is 25% for the load mode I, 20.8% for mode II, 20.5% for mode III, and 35.7% for mode IV.
(4) During the monitoring of the stress-strain state of the surface using the ODIC method, it is necessary to take into account the inhomogeneity of the stress-strain state along the thickness of the structural element. As the results of this work show, at a safe level of the surface layers’ strain on a structural element, a fracture can already begin inside it.
(5) Authors plan to conduct fractographic studies of the microstructural fractures of samples under different loading modes of hydrogenation in order to establish the influence of hydrogen and prestrain on the metal microstructure.

## Figures and Tables

**Table 1:** The value of the fracture energy in the centre and surface of the sample for the 4 load modes.

Area in the Sample	Fracture Energy (W_C_), MJ/m^3^			
	Load Mode I	Load Mode II	Load Mode III	Load Mode IV
centre	809	441	239	554
edge	607	349	190	356

## Data Availability

Not applicable.
